# Pneumococcal Meningitis in Adults after Introduction of PCV7 and PCV13, Israel, July 2009–June 2015^1^

**DOI:** 10.3201/eid2407.170721

**Published:** 2018-07

**Authors:** Gili Regev-Yochay, Klaris Reisenberg, Michal Katzir, Yonit Wiener-Well, Galia Rahav, Jacob Strahilevitz, Valery Istomin, Evgenia Tsyba, Avi Peretz, Shirley Khakshoor, Ron Dagan, on behalf of the Israeli Adult Invasive Pneumococcal Disease Group

**Affiliations:** Sheba Medical Center, Ramat Gan, Israel (G. Regev-Yochay, G. Rahav, S. Khakshoor);; Tel Aviv University, Tel Aviv, Israel (G. Regev-Yochay, M. Katzir, G. Rahav);; Soroka Medical Center, Beersheba, Israel (K. Reisenberg);; Bar-Ilan University, Safed, Israel (K. Reisenberg, A. Peretz);; Meir Medical Center, Kfar Saba, Israel (M. Katzir);; Shaare Zedek Medical Center, Jerusalem, Israel (Y. Wiener-Well);; Hadassah-Hebrew University, Jerusalem (J. Strahilevitz);; Hillel Yaffe Medical Center, Hadera, Israel (V. Istomin);; Barzilai Medical Center, Ashkelon, Israel (E. Tsyba);; Baruch Padeh Medical Center, Tiberias, Israel (A. Peretz);; Ben-Gurion University of the Negev, Beersheba (R. Dagan)

**Keywords:** meningitis, *S. pneumoniae*, *Streptococcus pneumoniae*, adults, pneumococcal conjugate vaccine, indirect effects, serotype replacement, PCV7, PCV13, Israel, invasive pneumococcal disease, bacteria, vaccine, streptococci, pneumococcal meningitis, nationwide surveillance

## Abstract

The indirect effect of pneumococcal conjugate vaccine on adult pneumococcal meningitis has not been thoroughly investigated. We present data from active surveillance on pneumococcal meningitis in adults in Israel occurring during July 2009–June 2015. Pneumococcal meningitis was diagnosed for 221 patients, 9.4% of all invasive pneumococcal disease (IPD) cases. Although overall IPD incidence decreased during the study period, meningitis increased nonsignificantly from 0.66 to 0.85 cases/100,000 population. Incidence of vaccine type (VT) pneumococcal meningitis (VT13) decreased by 70%, but non-VT13 pneumococcal meningitis increased from 0.32 to 0.75 cases/100,000 population (incident rate ratio 2.35, 95% CI 1.27–4.35). Pneumococcal meningitis patients were younger and healthier than nonmeningitis IPD patients, and 20.2% had a history of previous head surgery or cerebrospinal fluid leak compared with <2.0% of nonmeningitis patients (p<0.0001). Non-VT13 types that rarely cause IPD (15B/C, 6C, 23A, 23B, 24F) seem to be emerging as common causes of meningitis.

*Streptococcus pneumoniae* is the leading cause of bacterial meningitis for persons of all ages (*1*). Pneumococcal meningitis is a relatively rare but the most severe form of invasive pneumococcal disease (IPD). Untreated pneumococcal meningitis usually leads to death, and even with optimal treatment, mortality rates are high and disease is severe with frequent long-term sequelae (*1*,*2*).

Since the introduction of pneumococcal conjugate vaccines (PCVs) into the national immunization plans (NIPs) for children in different countries, IPD incidence has declined, not only among children but also among unvaccinated adult populations through herd (indirect) protection (*3*–*6*). Despite non-VT strains nearly completely replacing VT strains as the causative agents of invasive nasopharynx disease, this replacement by non-VT strains was only partial in both the pediatric and adult populations, presumably because of the lower invasive potential of most non-VT strains (*7*). However, a higher magnitude replacement of the serotypes associated with invasive disease was observed among immunocompromised and elderly populations than among the rest of the general population (*4*,*8*). Although several studies have assessed the effect of PCV on meningitis in children (*9*–*11*), data on the indirect effects on adult pneumococcal meningitis are scarce. The available data mostly address the effect of the 7-valent PCV (PCV7) (*12*,*13*) or PCV10 on pneumococcal meningitis in adults (*14*), and only 2 studies addressed the effect of PCV13 (*15*,*16*).

In Israel, pneumococcal polysaccharide vaccine 23 (PPSV23) has been part of the NIP for adults for many years; the constant coverage rate is >70% for adults >65 years of age. Administration of PCV7 at 2, 4, and 12 months of age and a catch-up plan was introduced to the NIP for children in July 2009, resulting in >70% vaccine coverage (>2 doses) for children <2 years of age within 1 year of implementation (*17*). Starting in October 2010, administration of PCV13 began to replace that of PCV7, and immunization coverage rapidly reached ≈95% for 2 doses and ≈90% for 3 doses (*18*). We previously described the indirect effect of PCV on the adult population in Israel and reported an ≈20% decrease of overall IPD incidence 4 years after the introduction of PCV7 and 2.5 years after the introduction of PCV13; we also reported that the proportion of pneumococcal meningitis cases among all IPD cases increased and that incidence did not decrease as did other IPDs (*4*). In this article, we assess meningitis IPD and nonmeningitis IPD incidence and the change in associated serotypes in adults 6 years after PCV7 introduction.

## Materials and Methods

### Ethics Statement

This study was conducted after protocols were approved by the Sheba Medical Center Institutional Review Board (Ramat Gan, Israel) and the Soroka University Medical Center Institutional Review Board (Beersheba, Israel). Because this study was a retrospective observational study, the institutional review boards waived the need for written informed consent, so informed consent was not obtained from participants. Therefore, all patient records and information were deidentified before analysis.

### Study Period and Population

The period evaluated was July 1, 2009–June 30, 2015, a 6-year period starting when PCV7 was introduced into the NIP for children. We included all culture-confirmed IPD patients of the adult (>18 years of age) population in Israel (n = 5,029,600 in 2009; n = 5,504,900 in 2015; http://www.cbs.gov.il).

### Surveillance System and Study Design

To ensure both a high rate of reporting and data collection from the medical records, a large research network named the Israeli Adult Invasive Pneumococcal Disease (IAIPD) group was established. This group includes 2 researchers from each of the 27 acute care hospitals of Israel: a researcher from the microbiology laboratory and an infectious disease physician devoted to following the IPD patients and collecting the required data, as described previously (*4*).

In Israel, all invasive *S. pneumoniae* isolates are required by law to be reported and sent to the Ministry of Health reference laboratory (Jerusalem, Israel), and several different methods were used by clinical staff members to collect these bacterial isolates before submission. In addition to this passive surveillance, active surveillance was performed involving a capture–recapture method, in which the IAIPD representative at each of the 27 laboratories performing blood and cerebrospinal fluid (CSF) cultures reported all invasive *S. pneumoniae* isolates on a weekly basis and transported them to study headquarters (Pediatric Infectious Disease Laboratory, Soroka University Medical Center), as described previously (*18*).

IAIPD investigators retrospectively collected data for every laboratory-identified case from the medical files. Data were available from 24 of the 27 centers, constituting 90.9% of all IPD cases that were identified. Data collected included sociodemographic data (sex, age, place of birth, city of residence); medical history, including concurrent medical conditions and IPD-predisposing medical conditions; substance abuse; smoking history; influenza in the days preceding hospitalization; and vaccination history for influenza and pneumococcal pneumonia. The Centers for Disease Control and Prevention definition for IPD-predisposing medical conditions, on which vaccination recommendations are based (https://www.cdc.gov/vaccines/vpd/pneumo/downloads/pneumo-vaccine-timing.pdf), was used to divide adults into 3 categories: 1) high-risk patients, defined as patients having chronic renal failure, HIV, medically induced or innate immunodeficiencies, asplenia, hematologic or metastatic malignancies, CSF leak, or prior neurosurgery; 2) at-risk patients, defined as patients with diabetes mellitus, congestive heart failure, chronic lung disease, cirrhosis, or an addiction to alcohol; and 3) not at-risk patients, defined as those who were not recognized as having a predisposing medical condition. According to the NIP for adults in Israel, it is recommended that high-risk patients be vaccinated with PCV13 and PPSV23 and at-risk patients with PPSV23. We also collected data on in-hospital complications: septic shock, need for ventilation, disability as determined by transition to a long-term care facility, and concurrent medical conditions.

### Case Definition

We defined IPD cases on the basis of positive pneumococcal cultures and did not include PCR testing or antigen detection, as described previously (*4*). We defined pneumococcal meningitis patients as those with an *S. pneumoniae*–positive CSF culture or those with an *S. pneumoniae*–positive blood culture who were also given a clinical diagnosis of meningitis by the treating physician (i.e., given a discharge diagnosis code of meningitis).

### Vaccination Policy and Vaccine Uptake

PCV7 was licensed in Israel in 2007 and was introduced into the NIP for children in July 2009 with a 2-, 4-, and 12-month schedule and a 2-dose catch-up plan for all children <2 years of age at the time of introduction. The methods used to evaluate vaccine uptake initiated in July 2009 are described elsewhere (*18*).

### Laboratory Testing

Susceptibility testing of isolates was performed at the local laboratory of each medical center. All centers assessed susceptibility to penicillin and ceftriaxone, including MIC determination by using ETEST (bioMérieux, Marcy l’Etoile, France), following the Clinical and Laboratory Standards Institute guidelines (http://www.facm.ucl.ac.be/intranet/CLSI/CLSI-2017-M100-S27.pdf). Serotyping was performed with all viable isolates at the headquarters laboratory by using the Quellung reaction (Staten Serum Institute, Copenhagen, Denmark).

### Statistical Analyses

We determined the denominators for calculating the incidence and mortality rates by using data from the Israeli Central Bureau of Statistics (http://www.cbs.gov.il). We calculated incidence rate ratios (IRRs) by dividing the incidence rate (IR) of year 6 by the IR of year 1. We calculated 95% Poisson CIs for IRs and IRRs by following the method of Greenland and Rothman (*19*). We performed a Poisson regression to test the trend in IRs over the 6-year study. We used multivariate logistic models to determine the predictors of death among IPD patients and the predictors of meningitis development among meningitis patients who were not at risk for IPD. In the pneumococcal meningitis mortality rate model, we included the following variables: age, sex, predisposing medical conditions, nonhematologic metastatic malignancies, smoking history, and source of infection. In the models tested to define predictors of meningitis development among not at-risk patients, we included the following variables: age, concurrent medical conditions (other than those known to be predisposing for IPD), and serotype. We grouped serotypes by vaccine coverage in 1 model and specifically tested serogroup 23 in another model. We presented the model with the higher goodness-of-fit (model with serogroup 23 serving as the predictor). We included predictor variables with p values <0.2 in the univariate analysis in the respective multivariate model. We calculated adjusted odds ratios, 95% CIs, and adjusted p values and used SAS 9.4 software (SAS Institute, Cary, NC, USA). We calculated the Simpson diversity index to assess the change in diversity of serotypes in the bacterial population (*20*).

## Results

During the 6-year study period, 2,579 IPD cases occurred, but 234 were excluded because of a lack of medical file data; 221 pneumococcal meningitis diagnoses in persons >18 years of age were reported, constituting 9.4% of all the IPD cases with diagnosis data available (n = 2,345). Of the 221 patients with pneumococcal meningitis diagnoses, *S. pneumoniae* was isolated from both CSF and blood cultures in 99 (44.8%) episodes, blood cultures only in 89 (40.3%), and CSF only in 33 (14.9%).

**Figure 1:**
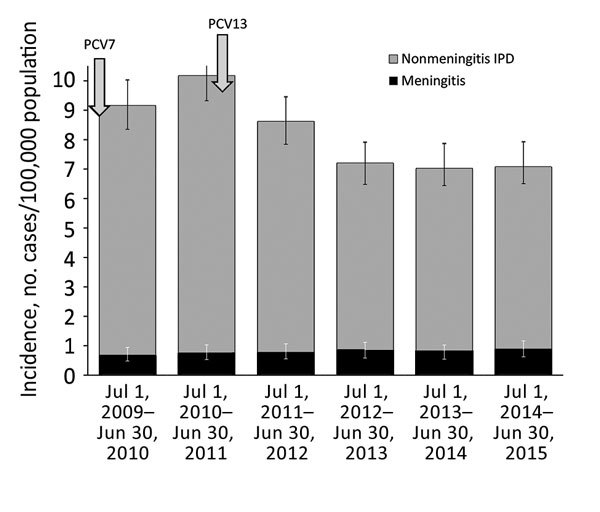
Incidence of meningitis and nonmeningitis IPD in patients >18 years of age, Israel, July 1, 2009–June 30, 2015. Introduction of PCV7 and PCV13 into the pediatric national immunization plan are depicted with arrows; 95% Poisson CIs are depicted for overall IPD and meningitis IPD. IPD, invasive pneumococcal disease; PCV, pneumococcal conjugate vaccine.

The mean annual incidence of pneumococcal meningitis was 0.77 cases/100,000 population, rising from 0.66 cases/100,000 population in the first year to 0.85 cases/100,000 population in the last study year, representing a 29% nonsignificant increase (IRR 1.29, 95% CI 0.81–2.06) (Figure 1; Technical Appendix Table 1). Over the same period, the incidence of nonmeningitis IPD decreased significantly by 26.7%, from 8.49 cases/100,000 population to 6.22 cases/100,000 population (IRR 0.73, 95% CI 0.63–0.85).

### Characteristics of Pneumococcal Meningitis Patients

Similar to other IPD patients, 75% of meningitis IPD patients were of Jewish ethnicity, and ≈50% were men (similar to the distribution in the general population) **(**Table 1**)**. Patients with pneumococcal meningitis were significantly younger than IPD patients without meningitis (p<0.0001). Moreover, concurrent medical conditions were less frequent among the meningitis IPD patients than the nonmeningitis IPD patients; 87.3% of meningitis patients and 92.8% of nonmeningitis patients (p = 0.004) had any concurrent medical condition, and 58.7% of meningitis patients and 68.6% of nonmeningitis patients (p = 0.003) were at risk or at high risk for IPD (had concurrent medical conditions for which PPSV23 is recommended). The most common high-risk concurrent medical condition among the pneumococcal meningitis patients, occurring in 20.2% of the population, was previous neurosurgery or CSF leak, which only occurred in 2.0% of the nonmeningitis IPD patients (p<0.0001).

**Table 1:** Univariate analysis of the characteristics of patients with meningitis IPD and nonmeningitis IPD by IPD source, Israel, July 1, 2009–June 30, 2015*

Variable	**Meningitis IPD, n = 221**	**Nonmeningitis IPD, n = 2,124†**				**p value§**
Pneumonia	Bacteremia, no source	IPD, rare types‡	All
Population with full data available	213	1,596	301	203	2,124	
Sex¶						
M	104 (48.6)	890 (55.8)	171 (56.8)	108 (53.2)	1,182 (55.7)	**0.047**
F	110 (51.4)	704 (44.2)	130 (43.2)	95 (46.8)	940 (44.3)	
Jewish ethnicity¶	166 (75.5)	1,157 (72.6)	221 (73.7)	154 (75.9)	1,551 (73.0)	0.46
Age, y¶						**<0.0001**
Mean	57.68	64.83	61.83	64.03	64.37	
Median (range)	61.47 (18.2–91.3)	66.53 (18.1–105.0)	65.45 (18.1–98.3)	66.91 (18.1–97.8)	66.35 (18.1–105.0)	
18–49	72 (32.6)	368 (23.1)	74 (24.6)	45 (22.2)	491 (23.1)	
50–64	68 (30.8)	382 (23.9)	75 (24.9)	47 (23.2)	510 (24.0)	
>65	81 (36.7)	846 (53.0)	152 (50.5)	111 (54.7)	1,123 (52.9)	
Concurrent medical conditions						
Any	186 (87.3)	1,481 (92.8)	287 (95.4)	189 (93.1)	1,972 (92.8)	**0.004**
High risk and at risk	130 (61.0)	1,093 (68.5)	233 (77.4)	156 (76.49)	1,495 (70.4)	**0.005**
High risk	81 (38.0)	645 (40.4)	179 (59.5)	107 (52.7)	939 (44.2)	0.083
Any immunosuppression	16 (7.5)	187 (11.7)	61 (20.3)	35 (17.2)	285 (13.4)	**0.014**
Bone marrow transplantation	4 (1.9)	42 (2.6)	17 (5.7)	12 (5.9)	71 (3.3)	0.248
HIV	0	33 (2.1)	3 (1.0)	3 (1.5)	39 (1.8)	**0.046**
Hematologic malignancy	19 (8.9)	208 (13.0)	68 (22.6)	41 (20.2)	320 (15.1)	**0.015**
Metastatic cancer	3 (1.4)	73 (4.6)	34 (11.3)	8 (3.9)	116 (5.5)	**0.010**
Asplenia	9 (4.2)	26 (1.6)	18 (6.0)	14 (6.9)	58 (2.7)	0.213
Chronic renal failure	14 (6.6)	277 (17.4)	46 (15.3)	35 (17.2)	363 (17.1)	**<0.0001**
Previous neurosurgery, CSF leak	43 (20.2)	29 (1.8)	6 (2.0)	7 (3.5)	42 (2.0)	**<0.0001**
At risk	49 (23.0)	448 (28.1)	54 (17.9)	49 (24.1)	556 (26.2)	0.314
Congestive heart failure	13 (6.1)	270 (16.9)	38 (12.6)	36 (17.7)	350 (16.5)	**<0.0001**
Chronic lung disease	20 (9.4)	358 (22.5)	35 (11.6)	29 (14.3)	425 (20.0)	**0.0002**
Cirrhosis	2 (0.9)	29 (1.8)	16 (5.3)	17 (8.4)	62 (2.9)	0.091
Diabetes mellitus	47 (22.1)	437 (27.4)	87 (28.9)	54 (26.6)	584 (27.5)	0.089
Alcohol abuse	3 (1.4)	51 (3.2)	10 (3.3)	8 (3.9)	69 (3.3)	0.138
Healthy	27 (12.7)	115 (7.2)	14 (4.7)	14 (6.9)	152 (7.2)	**0.004**

*Values are no. (%) except as indicated. CSF, cerebospinal fluid; IPD, invasive pneumococcal disease. †The source of 24 nonmeningitis IPD cases was not reported, so these cases were only included in the “All” column. ‡These IPD cases occurred after endocarditis, sinusitis, mastoiditis, peritonitis, endometritis, osteomyelitis, cellulitis, or septic arthritis; a few resulted from abscesses in various locations. §p value of meningitis vs. nonmeningitis IPD cases. Bold indicates significance. ¶The denominators are the total number of cases with information available pertaining to the relevant variable.

### Outcomes

Only in-hospital outcomes were available. The crude overall case-fatality rate was 15.5% for meningitis IPD patients and 23.0% for nonmeningitis IPD patients (p = 0.0123) (Table 2). After adjusting for age, risk group, and VT in a multivariate logistic model, we found that the mortality rate of patients with pneumococcal meningitis did not differ from that of patients with nonmeningitis IPD (Table 3). Other outcomes were worse for pneumococcal meningitis patients; compared with nonmeningitis IPD patients, meningitis IPD patients were more frequently hospitalized in the intensive care unit, more frequently required mechanical ventilation, and had longer lengths of hospital stay (median 6 days for nonmeningitis IPD vs. 15 days for meningitis IPD; Table 2). In addition, nearly 20% of meningitis IPD patients were discharged to long-term care facilities compared with 7% of nonmeningitis IPD patients.

**Table 2:** Univariate analysis of clinical outcomes of meningitis versus nonmeningitis IPD patients, Israel, July 1, 2009–June 30, 2015*

Category	Pneumococcal meningitis, n = 221	Nonmeningitis IPD, n = 2,124	p value
Population with full data available	213	2,123	
Overall no. deaths (case-fatality rate)	33 (15.5)	488 (23.0)	0.0123
ICU admission	115 (54.0)	322 (15.2)	<0.0001
Ventilation required	91 (42.7)	405 (19.1)	<0.0001
Mean LOS, d†	21.0	10.5	<0.0001
Percentile LOS, by days, %			
5	55	33	
10	38.5	21	
25	21	11	
50	15	6	
75	12	4	
90	10	2	
Sepsis†	10/180 (5.6)	170/1,635 (10.4)	0.0391
Discharged to long-term care facility†	34/173 (19.7)	104/1,490 (7.0)	<0.0001

*Values are no. (%) except as indicated. ICU, intensive care unit; IPD, invasive pneumococcal disease; LOS, length of stay. †Outcome assessed only for those who were discharged (deaths were excluded).

**Table 3:** Multivariate logistic model for predictors of death among IPD patients, Israel, July 1, 2009–June 30, 2015*

Variable	Adjusted OR (95% CI)	Adjusted p value
Age, y		
18–49		Referent
50–64	2.064 (1.39–3.06)	0.0003
>65	4.847 (3.42–6.87)	<0.0001
Risk group		
Not at risk		Referent
At risk	1.338 (0.99–1.80)	0.0561
High risk	1.609 (1.23–2.11)	0.0006
Serotype by VT		
VT7		Referent
VT13–7	0.903 (0.65–1.25)	0.539
Non-VT13	1.119 (0.84–1.50)	0.449
Nonmeningitis IPD		Referent
Pneumococcal meningitis	0.747 (0.50–1.11)	0.153


*IPD, invasive pneumococcal disease; OR, odds ratio; VT, vaccine type.

### Antimicrobial Drug Resistance

**Figure 2:**
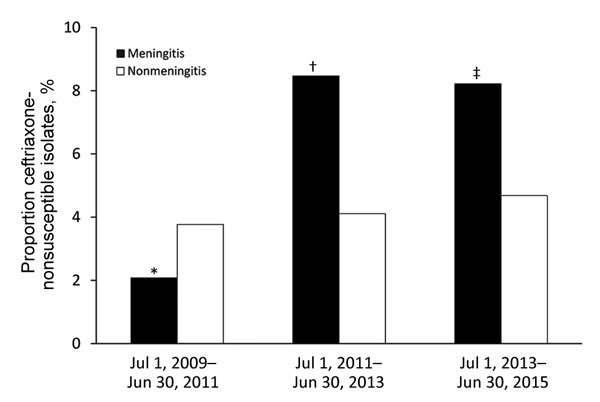
Proportion ceftriaxone-nonsusceptible isolates among all *Streptococcus pneumoniae* isolates acquired from patients with invasive pneumococcal disease, by 2-year period, Israel, July 1, 2009–June 30, 2015. Ceftriaxone-nonsusceptible isolates were those that could grow at a concentration above ceftriaxone’s MIC (>1 µg/mL). *In 2009–2011, the 1 ceftriaxone-nonsusceptible isolate was serotype 14. †In 2011–2013, the 5 ceftriaxone-nonsusceptible isolates included 2 of serotype 19F and 1 each of serotypes 19A, 34, and 23B. ‡In 2013–2015, the ceftriaxone-nonsusceptible isolates were serotypes 23F (n = 2) and 19A (n = 3), and 1 was not typeable.

Among the 221 isolates from meningitis IPD patients, 211 were susceptible to penicillin and 180 were susceptible to ceftriaxone. Isolates resistant to penicillin (MIC >0.06 µg/mL) were isolated from 25.1% of all meningitis patients. Ceftriaxone nonsusceptibility (MIC >1 µg/mL) emerged during the study years, from 2.1% (n = 1) in the first 2 years to >8% (n = 6) in the last 2 years (Figure 2). The single ceftriaxone-nonsusceptible meningitis isolate found during the first 2 years was serotype 14, but in the last 2 years, most ceftriaxone-nonsusceptible isolates were serotypes 19A and 23F. A single isolate of serotype 23F was highly resistant to ceftriaxone (MIC 2 µg/mL) and penicillin (MIC 8 µg/mL). The proportion of isolates from nonmeningitis IPD patients resistant to ceftriaxone, with a MIC >1 µg/mL (≈4%), did not change over the course of the study. Serotypes 14 and 19A each accounted for nearly 30% of the isolates, and 19F for 12%. All isolates were susceptible to vancomycin.

### Serotype Dynamics

**Figure 3:**
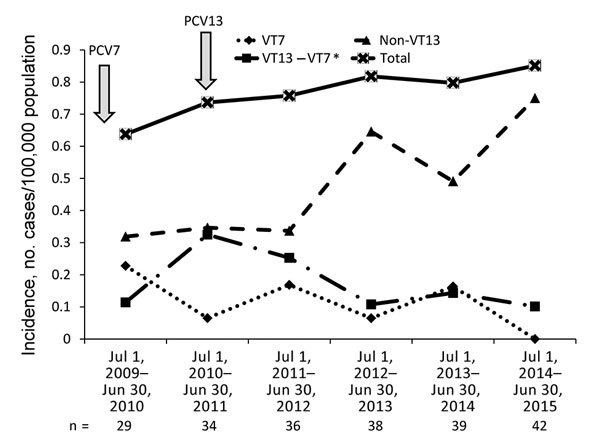
Incidence of pneumococcal meningitis in patients >18 years of age, by VT, Israel, July 1, 2009–June 30, 2015. The total number of cases per year are shown, and the introductions of PCV7 and PCV13 into the pediatric national immunization plan are depicted with arrows. *Serotypes included in the VT13 vaccine but not in the VT7 vaccine. PCV, pneumococcal conjugate vaccine; VT, vaccine type.

**Figure 4:**
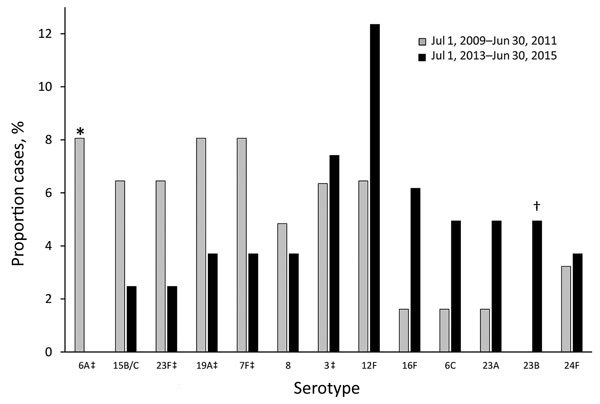
Comparison of serotypes causing pneumococcal meningitis during the first and last 2-year periods of study, Israel, July 1, 2009–June 30, 2011, and July 1, 2013–June 30, 2015. Only common serotypes (those occurring in >5% of cases in either the first 2-year period [n = 62] or last 2-year period [n = 81]) were included. *p<0.05; †p<0.1; ‡serotypes covered by pneumococcal conjugate vaccine 13.

Although overall pneumococcal meningitis incidence did not decrease, meningitis caused by VT13 serotypes decreased by 70% (IRR 0.297, 95% CI 0.11–0.82), similar to the decrease in VT13 nonmeningitis IPD cases. VT7 serotypes were totally eliminated by the sixth study year (Figure 3). The PCV13 serotypes 3, 7F, and 19A caused meningitis in the sixth study year and constituted 11.9% of all isolates that year. The diversity of the serotypes causing pneumococcal meningitis did not change substantially over the 6-year study; the Simpson diversity index was 0.951–0.974. To assess the dynamics of the serotypes commonly causing pneumococcal meningitis, we compared the serotype distribution of the first 2 study years (July 1, 2009–June 30, 2011; pre-PCV13 period) with that of the last 2 study years (July 1, 2013–June 30, 2015) (Figure 4). The dynamics of PCV13 serotype 3 contrasted with those of the other PCV13 serotypes; while the proportion and incidence of the common VT13 serotypes 6A, 23F, 19A, and 7F decreased, serotype 3 slightly increased. Despite the significant decrease in the incidence of VT13 strains and the elimination of VT7 strains, the overall incidence of pneumococcal meningitis increased because of a significant emergence of non-VT13 serotypes (IRR 2.352, 95% CI 1.27–4.35). The common emerging non-VT serotypes were 12F, 16F, 6C, 23A, 23B, and 24F.

**Figure 5:**
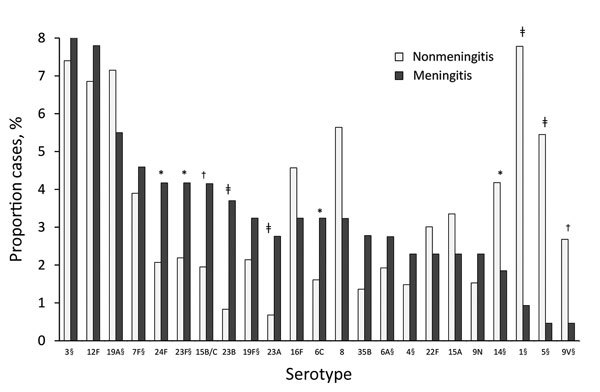
Serotypes associated with meningitis and nonmeningitis invasive pneumococcal disease, Israel, July 1, 2009–June 30, 2015. Only major serotypes (those totaling >3% of all *Streptococcus pneumoniae* isolates from all study years) were included. *p<0.1; †p<0.05; ‡p<0.005; §serotypes covered by pneumococcal conjugate vaccine 13.

Certain serotypes made up a significantly higher proportion of meningitis IPD than nonmeningitis IPD cases, particularly 23A (p = 0.0018) and 23B (p = 0.0001) but also 24F (p = 0.054), 23F (p = 0.072), 15B/C (p = 0.035), and 6C (p = 0.084) (all but 23F being non-VT13 serotypes) (Figure 5; Technical Appendix Table 2). Other serotypes were commonly isolated from patients with nonmeningitis IPD and rarely detected in patients with meningitis IPD, particularly serotypes 14 (p = 0.093), 1 (p = 0.0002), 5 (p = 0.0013), and 9V (p = 0.045).

### Predictors of Pneumococcal Meningitis

To elucidate unrecognized risks or predictors for pneumococcal meningitis beyond neurosurgery or CSF leak, we separately assessed all patients with IPD categorized as not at risk for IPD (i.e., without any recognized concurrent medical condition for which PPSV23 is recommended). First, we performed a univariate analysis comparing characteristics of the not at-risk patients with pneumococcal meningitis with those of the not at-risk patients with nonmeningitis IPD (Technical Appendix Table 3). The medical conditions trauma, lipid disorders, and chronic or recurrent infections were more frequently reported for the meningitis IPD not at-risk population than the nonmeningitis IPD not at-risk population. Smoking and dementia were less frequently reported for the meningitis IPD patient population. To determine independent predictors for pneumococcal meningitis among the not at-risk patient population, we performed a multivariate logistic analysis. In general, having any concurrent medical condition was associated with nonmeningitis IPD, and the particular medical conditions lipid disorder, chronic or recurrent infections, and previous trauma were independently associated with meningitis IPD. Many of the chronic or recurrent infections reported included otitis media or sinusitis, but the numbers of these cases were too small for greater resolution (Technical Appendix Table 3). Because we observed that certain serotypes were particularly associated with pneumococcal meningitis, we tested several models to determine whether particular serotypes predicted meningitis IPD in the not at-risk group. The best-fit model found that serogroup 23 was an independent predictor of pneumococcal meningitis in this patient population; adjusted odds ratio was 5.43 (95% CI 2.01–14.70) (Table 4).

**Table 4:** Multivariate logistic model for predictors of meningitis among patients not at risk for IPD, Israel, July 1, 2009–June 30, 2015*

Variable	Adjusted OR (95% CI)	Adjusted p value
Age, y		
18–49		Referent
50–64	2.13 (1.16–3.91)	0.015
>65	1.90 (1.02–3.55)	0.042
Concurrent medical conditions		
None		Referent
Any†	0.48 (0.27–0.85)	0.011
No history of previous trauma		Referent
Previous trauma	9.78 (2.24–42.75)	0.002
No lipid disorder		Referent
Lipid disorder	2.73 (1.19–6.24)	0.017
No history of Infections		Referent
Recurrent or chronic infections‡	12.31 (3.86–39.22)	<0.0001
No history of dementia		Referent
Dementia	0.12 (0.02–0.91)	0.040
Serotype		
Non-23		Referent
23	5.43 (2.01–14.70)	0.0009

*Patients not at risk for IPD were defined as those without any recognized concurrent medical condition for which PPSV23 administration is recommended. IPD, invasive pneumococcal disease; OR, odds ratio; PPSV23, pneumococcal polysaccharide vaccine 23. †Any concurrent medical condition, excluding those for which PPSV23 is recommended. Patients with these conditions were not included in the not at-risk group. ‡Various types of recurrent or chronic infections, including chronic otitis media, sinusitis, recurrent cellulitis, chronic osteomyelitis, and tuberculosis.

## Discussion

In our nationwide study, we assessed the dynamics of the incidence of pneumococcal meningitis in adults after sequential implementation of PCV7 and PCV13 in children. Our data demonstrate that some features of pneumococcal meningitis differ significantly from those of nonmeningitis IPD. The pneumococcal meningitis patient population was younger and had less frequent and different concurrent medical conditions. Outcomes also differed, with meningitis IPD patients having a more frequent need for ventilation, intensive care treatment, and treatment in long-term care facilities after discharge and a longer length of hospital stay. Yet mortality rates and case-fatality rates did not differ between the meningitis and nonmeningitis IPD populations. Although this finding seems surprising, a decrease in the pneumococcal meningitis case-fatality rate with adjuvant corticosteroid treatment was reported in a multicenter study in Europe (*21*). Although we do not know the proportion of patients in our study treated with corticosteroids, this treatment is part of routine practice for treating meningitis patients in Israel.

Ceftriaxone nonsusceptibility increased during the study period. This finding was unexpected because the only nonsusceptible isolate in the pre-PCV13 era was VT7 serotype 14. Antimicrobial drug recommendations did not change in a way that could have stimulated the emergence of ceftriaxone-nonsusceptible isolates. The emerging ceftriaxone-nonsusceptible isolates belonged mainly to serogroups 19 and 23 or non-VT13 serotypes (34 and an unencapsulated isolate [nontypeable]). Previous studies reported initial declines in rates of IPD with antimicrobial drug–resistant *S. pneumoniae* after the introduction of PCV7 in children and adults. These declines were attributed to the reduction of VT7 serotypes, which constituted most of the resistant strains. This observation of increased antimicrobial drug resistance is particularly worrisome; with ceftriaxone-nonsusceptible isolates totaling 8.3% of meningitis IPD cases, adding vancomycin to first-line therapy (pending culture results) might be necessary. Most of the isolates not susceptible to ceftriaxone were from serotypes covered by PCV13; thus, the ceftriaxone-nonsusceptible VT13 strains might be eventually eliminated through vaccination efforts, and these high nonsusceptibility rates might eventually decline with time. Although the European Society of Clinical Microbiology and Infectious Diseases and the Infectious Diseases Society of America guidelines (*22*,*23*) suggest adding vancomycin if a high prevalence of nonsusceptibility to penicillins and cephalosporins is observed, the guidelines do not specify at which resistance prevalence the policy should be enacted. Our results indicate the need for continued surveillance of ceftriaxone nonsusceptibility rates among pneumococcal meningitis cases.

The effect of the PCV NIP for children on pneumococcal meningitis in adults has been studied infrequently and mostly in the pre-PCV13 era. A review summarizing the effect of PCV7 on pneumococcal meningitis incidence in Europe and the United States reported a large variation in the effects, ranging from a 137% increase to a 77% decrease; the effect on VT7 pneumococcal meningitis incidence was also diverse (ranging from a 43% increase to an 87% decrease) (*24*). Studies in the United States and the Netherlands reported reductions in overall pneumococcal meningitis in adults after PCV7 or PCV10 introduction (*13*,*14*). Data on the effects of PCV13 on pneumococcal meningitis are scarce and include only data on pediatric populations; results differed according to geographic region. No significant change in pediatric pneumococcal meningitis rates was reported in 2 studies (*9*,*10*), and a decrease in pneumococcal meningitis in children <2 years of age was reported in others (*22*,*25*).

We show that in contrast to the significant indirect effect observed on overall IPD and particularly bacteremic pneumococcal pneumonia (*4*,*26*,*27*), adult pneumococcal meningitis incidence did not decrease after PCV implementation. We show that the reason for this is an increase of pneumococcal meningitis incidence caused by non-VT13 serotypes. A significant replacement in carriage of less invasive non-VT13 serotypes in children occurred after the PCV13 introduction; these strains had higher rates of transmission to adults (*7*). But why these non-VT serotypes cause more meningitis than nonmeningitis IPD is unclear. Our data suggest roles of both the host and the pathogen; a higher proportion of patients with meningitis IPD had previous neurosurgery operations, CSF leak, and history of trauma, during which direct penetration of carried bacteria could potentially occur and explain the greater replacement with non-PCV13 serotypes. We also found that odds of pneumococcal meningitis development was higher among patients with a lipid disorder and lower among patients with dementia. We have no explanations for these findings. The latter observation could potentially be explained by classification bias, if less diagnostic efforts were taken to understand the status of patients with dementia.

We also show that independent of concurrent medical conditions, several serotypes, mostly non-VT13 serotypes, were significantly associated with meningitis, suggesting a specific role of the pathogen in patients with concurrent medical conditions. Serotypes 6C and 23 are either directly covered by PCV13 or indirectly covered through cross-protection from antibodies developed against serotypes 6B and 23F. The only previous study of the indirect effects of PCV13 childhood vaccination on adult pneumococcal meningitis reported similar results as ours; however, these results were based on a small sample size (<20 cases of pneumococcal meningitis) (*16*).

This study has several limitations. First, data from the pre-PCV7 era were not collected. Second, antimicrobial drug susceptibility testing was performed in different laboratories and variations in testing might have occurred. Third, meningitis cases that were diagnosed on the basis of PCR without culturing were not included, and thus, meningitis rates might be underestimated. Last, this study has the limitations of a retrospective study; thus, only data available from the electronic file were collected.

In conclusion, this nationwide study indicates that PCV7 and PCV13 childhood vaccination indirectly affected the incidence of adult pneumococcal meningitis. We report the elimination of the occurrence of VT7 serotypes among pneumococcal meningitis patients within 6 years after PCV7 implementation and 4.5 years after PCV13 implementation. Yet we report no decrease in overall IPD meningitis cases because of the significant emergence of several non-VT13 strains, particularly serotypes 6C, 12F, 16F, 23A, and 23B.

Technical Appendix Meningitis and nonmeningitis invasive pneumococcal disease (IPD) incidence and case serotype and a univariate analysis of characteristics associated with meningitis IPD among patients not at risk for IPD, Israel, July 1, 2009–June 30, 2015.
