# Supplementary material for: Pneumococcal Meningitis in Adults after Introduction of PCV7 and PCV13, Israel, July 2009–June 2015
Source: Emerg Infect Dis. 2018 Jul;24(7):1275–84. doi: 10.3201/eid2407.170721 (PMC6038733; doi:10.3201/eid2407.170721)
Supplement: Technical Appendix — Meningitis and nonmeningitis invasive pneumococcal disease (IPD) incidence and case serotype and a univariate analysis of characteristics associated with meningitis IPD among patients not at risk for IPD, Israel, July 1, 2009–June 30, 2015. [file 17-0721-Techapp-s1.pdf]

Article DOI: https://doi.org/10.3201/eid2407.170721

Pneumococcal Meningitis in Adults after Introduction of PCV7 and PCV13, Israel, July 2009–June 2015

Technical Appendix

Technical Appendix Table 1. Nonmeningitis and meningitis IPD cases and incidence, by year, age, and VT, Israel, July 1, 2009–June 30, 2015*

Category	July 1, 2009–June 30, 2010	July 1, 2010–June 30, 2011	July 1, 2011–June 30, 2012	July 1, 2012–June 30, 2013	July 1, 2013–June 30, 2014	July 1, 2014–June 30, 2015	IRR (95% CI)
IPD incidence, no. (cases/100,000 population)†	460 (9.15)	520 (10.16)	449 (8.61)	382 (7.19)	379 (7.01)	389 (7.07)	0.773 (0.68–0.88)
No. IPD cases with full data	417	470	412	343	344	359	
Nonmeningitis IPD incidence, no. (cases/100,000 population)	387 (8.49)	436 (9.42)	376 (7.85)	304 (6.37)	305 (6.22)	316 (6.22)	0.733 (0.63–0.85)
Meningitis IPD incidence, no. (cases/100,000 population)	30 (0.66)	34 (0.73)	36 (0.75)	39 (0.82)	39 (0.79)	43 (0.85)	1.286 (0.81–2.05)
No. meningitis IPD cases with serotype data	29	34	36	38	39	42	
Meningitis IPD cases							
% VT7	34.5	8.8	22.2	7.9	20.5	0	
% VT13 – VT7†	17.2	44.1	33.3	13.2	18.0	11.9	
% non-VT13	48.3	47.1	44.4	79.0	61.5	88.1	
Meningitis IPD incidence by age, no. (cases/100,000 population)							
18–49 y	10 (0.346)	10 (0.339)	17 (0.574)	13 (0.429)	8 (0.257)	14 (0.425)	
50–64 y	7 (0.725)	8 (0.798)	11 (1.074)	14 (1.388)	16 (1.485)	12 (1.146)	
≥65 y	13 (1.965)	16 (2.395)	8 (1.101)	12 (1.646)	15 (1.988)	17 (2.103)	

*IPD, invasive pneumococcal disease; IRR, incidence rate ratio; VT, vaccine type.
†Refers to serotypes included in the VT13 vaccine but not in the VT7 vaccine.

Technical Appendix Table 2. Serotype distribution among meningitis and nonmeningitis IPD patients, Israel, July 1, 2009–June 30, 2015*

Serotype	Meningitis IPD, N = 217, no. (%)	Nonmeningitis IPD, N = 2,055, no. (%)	OR (95% CI)
19A	12 (5.5)	147 (7.2)	NS
24F	9 (4.2)	43 (2.1)	NS
15B/C	9 (4.2)	40 (2.0)	2.17 (1.04–4.53)
19F	7 (3.2)	44 (2.1)	NS
23F	9 (4.2)	45 (2.2)	NS
23A	6 (2.8)	14 (0.7)	4.13 (1.57–10.85)
16F	7 (3.2)	94 (4.6)	NS
6C	7 (3.2)	33 (1.6)	NS
23B	8 (3.7)	17 (0.8)	4.59 (1.96–10.76)
8	7 (3.2)	116 (5.6)	NS
35B	6 (2.8)	28 (1.4)	NS
14	4 (1.8)	86 (4.2)	NS
1	2 (0.9)	160 (7.8)	0.11 (0.03–0.45)
5	1 (0.5)	112 (5.5)	0.08 (0.01–0.58)
9V	1 (0.5)	55 (2.7)	0.17 (0.02–1.22)
3	18 (8.3)	152 (7.4)	NS
12F	17 (7.8)	139 (6.8)	NS
7F	10 (4.6)	81 (3.9)	NS
6A	6 (2.8)	41 (2.0)	NS
4	5 (2.3)	31 (1.5)	NS
22F	5 (2.3)	62 (3.0)	NS
15A	5 (2.3)	69 (3.4)	NS
9N	5 (2.3)	31 (1.5)	NS

*IPD, invasive pneumococcal disease; NS, not significant; OR, odds ratio. Serotypes presented are those that consisted of >3% of all isolates in all years.

Technical Appendix Table 3. Univariate analysis of characteristics associated with meningitis IPD among IPD patients not at risk for IPD, Israel, July 1, 2009–June 30, 2015*

Characteristic or risk factor	Meningitis IPD, N = 83, no. (%)	Nonmeningitis IPD, N = 629, no. (%)	p value†
Age, y			
Mean ± SD	57.9	56.1	**0.014**
Median	61.5	56.4	
<50	29 (34.9)	283 (45.0)	
50–64	28 (33.7)	125 (19.9)	
≥65	26 (31.3)	221 (35.1)	
Healthy	27 (32.5)	152 (24.2)	0.099
Smoker	10 (12.1)	132 (21.0)	**0.056**
Concurrent medical condition			
Malignancies	4 (4.8)	66 (10.5)	0.103
Dementia	1 (1.2)	54 (8.6)	**0.018**
Trauma	4 (4.8)	5 (0.8)	**0.002**
Debilitated	0	5 (0.8)	0.415
Neurologic disease	8 (9.6)	44 (7.0)	0.384
CVA	1 (1.2)	18 (2.9)	0.379
IHD	1 (1.2)	16 (2.5)	0.453
Lipid disorder	10 (12.1)	36 (5.7)	**0.028**
Chronic or recurrent infectious disease‡	8 (9.6)	6 (1.0)	**<0.0001**
Pneumococcal isolate serotype			
VT7	9 (10.8)	71 (11.6)	0.839
23F	4 (4.8)	10 (1.6)	**0.049**
19F	1 (1.2)	9 (1.5)	0.858
14	1 (1.2)	21 (3.4)	0.283
4	1 (1.2)	10 (1.6)	0.778
18C	1 (1.2)	4 (0.7)	0.569
9V	1 (1.2)	11 (1.8)	0.706
6B	0	6 (1.0)	0.368
VT13 – VT7§	26 (31.3)	286 (46.7)	**0.008**
3	10 (12.0)	44 (7.2)	0.112
19A	7 (8.4)	36 (5.9)	0.349
7F	6 (7.2)	42 (6.9)	0.879
1	1 (1.2)	92 (15.0)	**<0.001**
5	1 (1.2)	61 (10.0)	**0.009**
6A	1 (1.2)	11 (1.8)	0.706
Non-VT13	48 (57.8)	255 (41.7)	**0.005**
12F	9 (10.8)	49 (8.0)	0.362
15B/C	6 (7.2)	3 (0.5)	**<0.001**
24F	4 (4.8)	8 (1.3)	**0.020**
8	4 (4.8)	57 (9.3)	0.175
16F	3 (3.6)	18 (2.9)	0.722
6C	3 (3.6)	7 (1.1)	**0.073**
23A	2 (2.4)	3 (0.5)	**0.052**
23B	2 (2.4)	3 (0.5)	**0.050**

*Patients not at risk for IPD were defined as those without a concurrent medical condition known to put them at risk for IPD. CVA, cerebrovascular accident; IHD, ischemic heart disease; IPD, invasive pneumococcal disease; VT, vaccine type.
†Significant p values are in boldface.
‡Includes patients with recurrent cellulitis, chronic sinusitis, chronic or recurrent otitis media, chronic osteomyelitis, and history of tuberculosis.
§List includes serotypes in the VT13 vaccine but not in the VT7 vaccine.
